# Psychosomatic–psychotherapeutic treatment in an evening clinic: a qualitative examination of patients’ expectations and experiences

**DOI:** 10.1186/s13033-019-0326-3

**Published:** 2019-11-07

**Authors:** F. Brunner, U. Dinger, M. Komo-Lang, H. C. Friederich, H. Schauenburg, W. Herzog, C. Nikendei

**Affiliations:** Department of General Internal Medicine and Psychosomatics, Centre for Psychosocial Medicine, University Hospital Heidelberg, Thibautstrasse 4, 69115 Heidelberg, Germany

## Abstract

**Background:**

Over a course of 10 weeks the psychosomatic–psychotherapeutic evening clinic at the University of Heidelberg offers an intensive and multimodal 3-h treatment program on three evenings a week. The clinic aims at accommodating patients who on the one hand do not fit the criteria of partial or full-time inpatient therapy, but on the other hand requires a more intensified therapy dose than the usual German outpatient settings can cater for. In the presented monocentric, qualitative study, we wanted to examine this treatment concept with regard to the patients’ specific concerns, expectations, and individual experiences. By contrasting differences in intensity of outpatient and inpatient treatment, we aimed to identify those characteristics of the evening clinic setting that were perceived as especially helpful.

**Method:**

Each of the 25 patients was interviewed twice, using semi-structured interviews. The interviews took place before (T0) and after (T1) the 10-week treatment interval. A qualitative content analysis of the transcribed interviews was performed using the software “MaxQDA”.

**Results:**

We identified a total of 1609 separate codes and grouped them into 33 topics and 5 overarching categories. Here, we found some aspects independent of the therapeutic setting, and others concerning the patients’ specific expectations and experiences resulting from the particularities of the evening clinic as an outpatient setting including certain inpatient characteristics. This included the possibility of patients continuing to work and being able to fulfil social obligations, i.e. childcare or caring for relatives, while at the same time undergoing intensive psychotherapeutic treatment.

**Conclusions:**

Our results show that the evening clinic concept is particularly suitable for patients with mental and psychosomatic disorders who require intensified multimodal therapy while continuing to meet their obligations in their private and working lives. However, in comparison to other therapeutic methods, this concept generated greater stress and time challenges. Patients should therefore have a reasonably good standard of functioning in everyday life and sufficient coping resources. This is especially important for patients who continue working in their jobs while undergoing treatment. So far, there is a lack of quantitative data which would be needed to evaluate the effectiveness of this novel setting.

## Background

In the treatment of psychosomatic–psychotherapeutic illnesses, the Fifth Social Code (SGB V) of the Federal Republic of Germany distinguishes between outpatient guideline psychotherapy on the one hand, and partial or full-time psychotherapy in hospitals on the other hand. As a general rule, statutory insurers cover the costs for psychotherapy for all mental illnesses and disorders for which treatment is indicated. In addition, if a physical illness causes considerable psychological strain, for example tinnitus or cancer often accompanied by depression, the health insurance company will cover the costs for psychotherapy in Germany. However, statutory health insurers do not cover the costs for all psychotherapy schools. To date, five approaches are guideline approved in Germany: analytical psychotherapy, depth psychology therapy, cognitive behavioural therapy, EMDR, and systemic psychotherapy. Nevertheless, a wide variety of different approaches can be used within these state approved approaches. The scope of outpatient psychotherapeutic care in Germany is regulated in the Psychotherapy Guidelines [1]. This Guideline serves to achieve an appropriate, adequate, and economic psychotherapy of the insured persons in Germany covered by statutory insurers. As a general rule, statutory insurers cover the costs for psychotherapy for all mental illnesses and disorders that are considered to need treatment. However, statutory health insurers do not cover the costs for all types of psychotherapy. There are currently three state approved approaches in Germany: analytical psychotherapy, depth psychology therapy, and cognitive behavioural therapy. Depth psychology therapy and cognitive behavioural therapy usually take place once a week with sessions lasting 50 min each, while the analytical setting can comprise two to three therapy sessions per week. Statutory health insurers cover up to 80 therapy sessions for behavioural therapy, up to 100 for depth psychology therapy, and up to 300 for analytical therapy [1]. The effectiveness of outpatient guideline therapy has been proven in several German studies [2–4] as well as internationally [5, 6].

Part-time or full-time inpatient psychotherapy programmes are based on an integrative, method- and school-spanning psychotherapeutic concept with a combination of different therapeutic approaches, for example group therapy settings, individual treatment, and family or couple therapy. Many clinics focus on group psychotherapy [7]. There are various indications for a patient to be in need of inpatient psychotherapy, such as the severity of symptoms, a high degree of impairment in everyday life, suicidality, pronounced psychosocial difficulties or domestic conflicts, as well as insufficient outpatient therapy offers or accessibility [8–10]. Several studies have shown the efficacy of inpatient treatment, especially in regard to the reduction of symptoms, frequency of physician contacts, and sick days [9–13].

Compared to guideline outpatient therapy, the advantages of (partial) inpatient therapy programs lie in higher treatment intensity and in the possibility of combining individual and group therapy offers more easily. Furthermore, both verbal and non-verbal therapy methods can be applied. Work absenteeism due to sick leave and removal from the patients’ home environment may bring further relief, especially for highly burdened patient groups with low current everyday functioning [14]. On the other hand, it is our experience, that the required absence from work or educational training, the removal from usual home environments and family obligations can result in a loss of self-esteem, self-efficacy, and supportive stabilization in some patients, which in turn can affect the overall success of therapy. Our long-term experience with patients seeking help in our psychosomatic outpatient clinic also shows, that professional or private obligations sometimes do not allow for longer hospital stays and some patients fear stigmatization both in their home and their working surroundings. Furthermore, many of our patients are concerned about the difficulties arising at their workplaces if they are absent for several weeks.

Our clinical experience with patients has shown that there is a gap between low-frequency outpatient guideline psychotherapy and the cost- and time-intensive multimodal inpatient psychotherapy programs in the German health care system. Besides, in the highly-regulated German health system, which only distinguishes between outpatient guideline psychotherapy on the one hand, and partial or full-time inpatient psychotherapy on the other hand, there is a need for innovative models and for corresponding projects that bridge the support gap between outpatient and inpatient psychotherapy [1].When inpatient treatment is disadvantageous, not possible or not necessary, there are no alternative treatment options for patients with intensive treatment needs. Especially for young people, online interventions, mainly based on a cognitive behavioral focus, offer an opportunity for mental health support that seem to be immediate and cost-effective [15]. In recent years, several studies have addressed the use of online interventions for the management of a number of mental disorders, with research supporting the efficacy of these interventions in alleviating anxiety and depressive symptoms [16]. Two Australian studies even report online interventions to be as effective as face-to-face therapy in depression an social phobia [15, 17], and one US-American randomized clinical trial recommends the use of online interventions within clinical guidelines for the treatment of depression [18]. However, most studies did not evaluate online-intervention against a competing intervention or control group. Furthermore, Rice et al. underline that the effect of online interventions very much depends on the participant attrition [19]. Indeed, in one US-American research only 19% of those potentially eligible patients enrolled [20] and studies promoting greater engagement of the participants tended to report lower attrition rates. Automated self-help services require significant motivation and self-discipline [21], which may be an enormous challenge for young people experiencing depression. The authors conclude, that ongoing engagement and high intervention adherence are important factors for the effect of online interventions [22].

Based on this point an our clinical observation, Heidelberg University Hospital now has developed a new, innovative model which combines the advantages of outpatient and inpatient treatment settings: a psychosomatic–psychotherapeutic evening clinic [23]. This novel setting aims to provide an intensive, multimodal psychotherapeutic offer while allowing patients to maintain and promote existing skills and coping strategies in their everyday lives. According to current literature, there are only a few models for psychosomatic–psychotherapeutic evening clinics worldwide. In Canada and the United States, especially, therapists are currently gathering experiences with this new treatment concept. Examples include the Evening Treatment Program at the Alberta Hospital, Edmonton, Alberta, Canada; and the Core Program of Richmond Mental Health Outpatient Services, Richmond, BC [24, 25]. In the latter program, naturalistic studies have shown positive effects on symptoms and the quality of life, interpersonal problems, and alexithymia [24, 26]. So far the effectiveness of the evening clinic model has yet to be systematically evaluated. The University of Heidelberg is currently investigating this aspect in an ongoing study. Further data were not available at this point. However, studies comparing the efficacy for day-clinic and inpatient psychotherapy show no difference between both settings [14, 27]. Only in terms of bulimia nervosa data suggest a slight advantage of day clinic treatment in long-term outcome [10].

So far, the expectations, concerns, and subjective experiences of patients being treated in an evening clinic setting have not been assessed. The current study aimed to investigate these questions qualitatively through semi-structured interviews in a pre-post-design. The main aim of the study was to investigate patients’ concerns, struggles, and experiences before and after being treated in the evening clinic. The secondary aim was to identify specific characteristics of the evening clinic setting that were perceived as helpful or difficult compared to other outpatient and inpatient treatment settings.

## Methods

### Study design

The study was conducted from March 2015 to July 2016 as a prospective, monocentric pilot study at the Department of General Internal Medicine and Psychosomatics of Heidelberg University Hospital. The semi-structured interviews took place before patients had started with (T0) and after they had completed their treatment in the evening clinic (T1).

### Study sample

We conducted semi-structured interviews with twenty-five patients, investigating their concerns and expectations prospectively (T0) and assessing their impressions and experiences retrospectively (T1). In addition, demographic data such as age, gender, occupational status, and diagnosis according to ICD-10 were collected and descriptively recorded (see Table 3). We included all patients who were at least 18 years old and were treated in our evening clinic setting. The evening clinic’s psychotherapy offer is directed at patients from the entire spectrum of psychological and psychosomatic illnesses with a focus on depression/burnout and anxiety disorders, as well as threshold-related psychological crises with relative stability prior to patients’ decompensation [23, 28]. In qualitative research the sample number can be determined gradually in the sense of a “theoretical sample” according to Glaser und Strauss [29]. This means that decisions on the selection and composition of empirical material in the process of data collection and evaluation depends on the results of the evaluation and the interests of the researcher until “theoretical saturation” is achieved [30]. Guest et al. were able to show that they had created 92% of the total number of codes developed for all thirty interviews conducted in their study after twelve interviews [31]. In our study with 25 participants, the “theoretical sample” according to Glaser und Strauss was reached.”. All participants were given details about the background of our study before taking part. Their participation was voluntary.

### The concept of the evening clinic

The Heidelberg evening clinic accommodates for eight patients suffering from mental or psychosomatic illness. The main focus lies on the treatment of patients with depression, anxiety disorders, and crisis in the context of threshold situations, such as difficulties in developing autonomy in adult life or familiar/social problems. The therapy is organized as a 10-week program and patients are treated in 3-h sessions on three evenings a week. This gives them the possibility of continuing with their professional and/or academic careers. Furthermore, patients can start or plan reintegration to their workplace after an absence while still continuing to attend the evening clinic. In line with the Göttinger model [32]; the therapeutic program combines psychoanalytic-interactional group therapy two sessions a week (one 60-min and one 90-min session), with 30 min of individual psychodynamic psychotherapy, a 60-min mindfulness group and a 15-min medical-psychotherapeutic doctor`s visit for therapy planning and evaluation. Additionally, each therapy day opens with a welcome round and check in which patients briefly state how they are doing and what they would like to discuss. Furthermore patient share communal dinner during treatment days and there is a 30-min mindfulness-focused closing session at the end of each treatment week [23]. The standard treatment duration is 10 weeks. The emphasis is placed on the concept of group therapy. Furthermore, the evening clinic offers the possibility of psychosocial or family-therapeutic elements as well as psychopharmacotherapy if necessary.

### Ethics

The study was conducted in accordance with the Declaration of Helsinki [33] and the study protocol was reviewed and approved by the Ethics Committee of the Department of Internal Medicine and Psychosomatic Medicine of the University of Heidelberg (S-013/2012). Participation in the study was voluntary. All patients received a detailed information sheet and gave their informed consent prior to participation in the study.

### Development of the interview guidelines

The study’s key questions and hypotheses for both interviews (T0 and T1) were developed in line with the criteria of the COREQ checklist on the basis of an in-depth literature review as well as discussion among a team of experts. The COREQ checklist is a 32-item checklist for explicit and comprehensive reporting of qualitative studies that aims to help reporting important aspects of the research team, study methods, context of the study, findings, analysis, and interpretations. The interviews were semi-structured [34–36], including open key questions which were followed by more focused questions. The key questions dealt with the patients’ prior and current concerns, as well as their impressions and experiences related to treatment in the evening clinic. The individual interviews were carried out under supervision by an experienced interviewer. All interviews were recorded by voice recorder and were later transcribed verbatim. The mean duration of the interviews was 52.03 ± 6.38 min (T0) and 51.44 ± 7.06 min (T1). The interview guidelines are listed in Tables 1 and 2.

**Table 1:** Interview guideline T0

Key question	Maintaining questions	Demands
First question: Expectations and hopes		
What expectations and hopes do you have concerning the evening clinic?	Can you describe this in more detail? Can you be a little more specific? Are there any more specific hopes or requests that you associate with the evening clinic?	–
Second question: Advantages		
What advantages do you expect from treatment in the evening clinic?	Can you describe this in more detail? Can you be a little more specific? Are there any specific benefits that you associate with the evening clinic?	Do you expect advantages over other treatment settings (outpatient, day-care, inpatient therapy)? Do you expect any benefits by continuing to be in your everyday environment, such as integrating issues that have been discussed in therapy into your private life? Do you see any benefits in limiting and intensifying the therapeutic program?
Third question: Problems and difficulties		
What problems and difficulties do you see in connection with treatment in the evening clinic?	Can you describe this in more detail? Can you be a little more specific? Are there any specific problems that you associate with the evening clinic?	Do you expect difficulties compared to other treatment settings (outpatient, day-care, inpatient therapy)? Are you experiencing difficulties in maintaining close ties to your everyday environment (work/study, family) during treatment in the evening clinic? Do you see any difficulties in limiting and intensifying the therapeutic program?
Fourth question: Disadvantages		
Do you see specific disadvantages that could arise when you attend the evening clinic?	Can you describe this in more detail? Can you be a little more specific? Are there specific problems that you associate with the evening clinic?	Do you see disadvantages compared to other treatment settings (outpatient, day-care, inpatient therapy)? Do you expect disadvantages regarding the fact that during treatment in the evening clinic you have a close relation to your everyday environment (job/study, family)? Do you see any disadvantages in limiting and intensifying the therapeutic program?

**Table 2:** Interview guideline T1

Key question	Maintaining questions	Demands
First question: Perception of the evening clinic		
How did you experience treatment in the evening clinic?	Can you describe this in more detail? Can you be a little more specific?	Are there special moments or experiences that remind you of the evening clinic?
Second question: Positive effects of the evening clinic		
What did you profit from?	Can you describe this in more detail? Can you be a little more specific?	Are there any more specific positive effects that you associate with the evening clinic? Were there certain situations that you found particularly helpful?
Third question: Difficult aspects of the evening clinic		
What did you find difficult?	Can you describe this in more detail? Can you be a little more specific?	Are there any more difficulties that you associate with the evening clinic? Were there certain situations that you found difficult or less helpful?

### Qualitative content analysis and quantitative descriptive statistics

We performed an open line by line coding of all 50 interviews to identify recurring topics. Subsequently, the T0 and T1 interviews were analysed separately. The qualitative content analysis was conducted according to Mayring’s qualitative content analysis criteria [35]. Using the software “MaxQDA” Version 11, Release 11.1.2., independent investigators first identified the information most pertinent to the posed question, as “codes” that represented the smallest units of meaning in the respective statement [37]. Second, names were given to each unit of information identified. Third, these content units were compared, ordered, and grouped until overarching relevant themes could be defined. In a final step, themes were summarized into five relevant categories. The descriptive data analysis for the characterization of the examined sample was carried out using the statistical program SPSS (IBM SPSS Statistics 20). Results were represented as mean ± standard deviation and, if possible, as median and quartile.

## Results

### Quantitative study sample

In total, 25 patients (60% male; mean age 40.5 ± 13.3 years) participated in our study. According to the criteria of the ICD-10, 22 patients (88%) suffered from a depressive disorder of varying severity and with different comorbidities: four (18%) had no other diagnosis and 18 (82%) had comorbid anxiety disorders, somatoform disorders or eating disorders. One patient (4%) suffered mainly from a somatoform disorder and one patient from an anxiety disorder. In total, four patients (16%) were diagnosed with a personality disorder in the sample (see Table 3). Of the interviewed patients, 11 continued to work full-time (44%) and three patients (12%) continued their studies without taking sick leave. During evening clinic treatment, four patients (16%) started planning their reintegration into their professional lives, whereas seven patients (28%) were unable to work for the entire duration of the treatment.

**Table 3:** Study sample

Parameters	Number
Age (years)	40.5 ± 13.3
Gender m:f (%)	15:10 (60:40)
Duration of treatment (days)	70 ± 26.1
Diagnoses	
Depression, n (%)	22 (88)
Anxiety disorder, n (%)	7 (28)
Eating disorder, n (%)	5 (20)
Somatoform disorder, n (%)	2 (10)
Personality disorder, n (%)	4 (16)
Professional situation	
Continuing to work n (%)	11 (44)
Continuing to study n (%)	3 (12)
Reintegration initiated n (%)	4 (16)
Incapacity to work n (%)	7 (28)

Y, years; m, male; f, female; d, days; n, number of patients. For patients with multiple mental diagnoses, each diagnosis was scored separately

### Main categories and topics

**Fig. 1:**
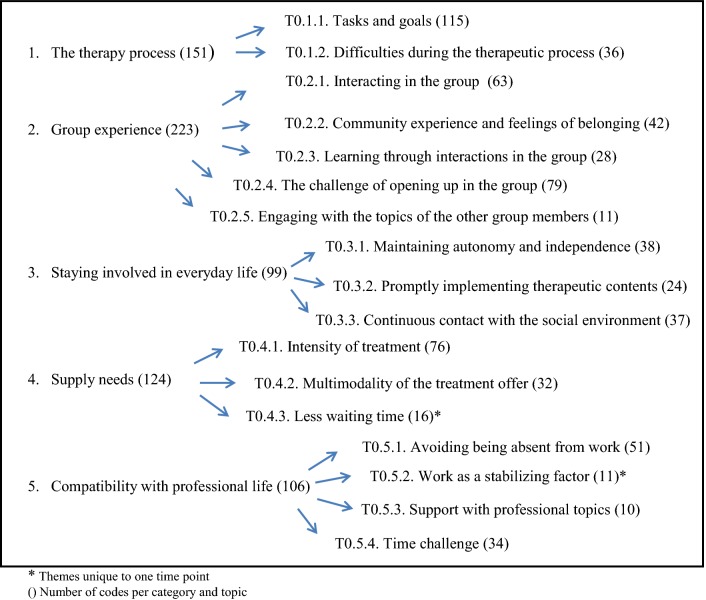
Flowchart listing the topics at T0 and their number of codes

**Fig. 2:**
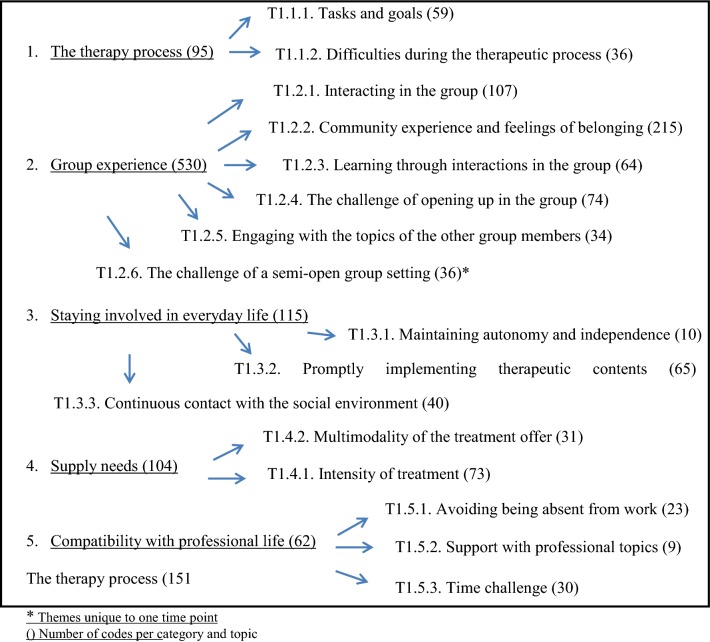
Flowchart listing the topics at T1 and their number of codes

The qualitative analysis of the interviews gave us a total of 1609 individual codes: 703 for the T0 interviews and 906 for the T1 interviews. These codes were then grouped into 33 topics which in turn were sorted according to five categories (Figs. 1, 2). In the following section the details of the topics and categories are listed in an overview and will be explained in detail later: The topics taken from the interviews before evening clinic treatment will be called T0.x.x. and the topics derived from the interviews after evening clinic treatment will be called T1.x.x. The number of codes per category and topic are shown in parentheses. Themes unique at one time point are marked by asterisk. Illustrative quotations for main categories and themes are listed in Tables 4, 5, 6, 7, and 8. The letter in parentheses behind the quotes represents the participant’s ID. For reasons of data security, randomly allocated letters were used and not the initial letters of the participants.

**Table 4:** Quotes on the therapy process before (T0) and after (T1) treatment

1. The therapy process
T0.1.1 Tasks und goals (115) ”Concerning my depression, I had hoped that my mood would generally improve and that I would be less anxious and could start processing everything.”(G) ”It is about processing. How to deal with certain situations or things that happen and to understand why my body or I react in a certain way—it is something completely new.”(Y) “The regular sessions encourage you to leave your comfort zone and talk about things. Rather than pushing everything away you have to engage with certain topics, more or less.”(H) T0.1.2 Difficulties during the therapeutic process (36) ”That it may not work, that I will be disappointed and have to look for another way—that would be awful.”(R) “That I have to confront myself with my anxieties, problems and assessments in front of the group. That is what I find difficult, too.”(S)
T1.1.1 Tasks and goals (59) “Yes, so, I’ve learned to cope better with thoughts or perhaps feelings, also in the moment.”(K) “And every time I was there I felt very safe, I always had this feeling: Nothing is going to happen to me here.”(U) T1.1.2 Difficulties during the therapeutic process (36) “I would have wished for more support in that direction.”(F)

**Table 5:** Quotes on group experience before (T0) and after (T1) treatment

2. Group experience
T0.2.1 Interacting in the group (63) “It’s the exchange with other patients who have had a similar fate or have made similar experiences. You get to learn about new perspectives that you haven’t encountered or haven’t been able to encounter before.”(F) T0.2.2 Community experience and feelings of belonging (42) “When you are not feeling well mentally, you are stronger in a group setting, I think. The incentive and the motivation are greater. Also, you receive help outside of the therapy sessions. You don’t know what kind of people you will meet there, perhaps you will find new friends. People, who can support you. I think it is really intensive to do group therapy together with someone.”(U) T0.2.3 Learning through interactions in the group (28) ”[…] … and you can get into contact with other people. On the one hand, in order to differentiate, on the other hand to set your boundaries and to engage with others, telling them that here is my space, my boundary, and there is the other person’s boundary. This would help interacting with other people.”(Q) T0.2.4 The challenge of opening up in the group (79) “You would like to be strong and not show any weakness, because that would make you seem vulnerable and could lead to others hurting you. And the more people know about it, the more difficult it gets. This is a disadvantage of the evening clinic because there you have to open up to a group of 6 people as well as to the medical professionals.”(F) “That I won’t be accepted into the group or that I will have conflicts within the group.”(S) T0.2.5 Engaging with the topics of the other group members (11) ”[…] that I might find it difficult to open up and deal with the problems of others.”(K)
T1.2.1 Interacting in the group (107) ”Yes, you may just see other courses of action or that people deal with them differently, other problem-solving possibilities are pointed out that you may not have thought of yourself yet.”(H) ”Yes, that several things have changed, that some things were different in the past, that I have become more open, that I am looking for more conversations, especially if something disturbs me, that I then speak about it, for example.”(F) T1.2.2 Community experience and feelings of belonging (215) ”And so it is good in this group that you see that everyone is struggling with these problems and that also creates the incentive to develop there.”(C) T1.2.3 Learning through interactions in the group (64) ”And then also the interaction in the group and with the other patients, that’s also something that I have a hard time with, with social things, social interaction and so that was actually such, yes, such a good test field for it.”(N) T1.2.4 The challenge of opening up in the group (74) ”Was it harder for me to open up, well, to talk about my problems because I thought the other -, the problems of the others are more important.”(R) T1.2.5 Engaging with the topics of the other group members (34) Because at that moment I pushed myself all, all the way back. And I didn’t know how to deal with other people’s problems either, so it must have taken me a week to even know how to deal with them.”(W) T1.2.6 The challenge of a semi-open group setting (36) “And that the group members were, uh, constantly changing, that made me really, uh, even harder.”(S)

**Table 6:** Quotes on staying involved in everyday life before (T0) and after (T1) treatment

3. Staying involved in everyday life
T0.3.1 Maintaining autonomy and independence (38) “Yes, actually I don’t feel so sick that I have the feeling I would need comprehensive care. I manage to deal with everyday stuff and I’m also quite content that I’m able to keep my flat in order.” (O) T0.3.2 Promptly implementing therapeutic contents (24) “The problem of going to hospital is that you will be staying there, you get “full board” and perhaps you also learn a lot there, but when you leave you suddenly—boom—have to cope with your everyday life. This is not the same with the evening clinic. When you have learnt something there, you can immediately try to include it in your daily life.”(I) T0.3.3 Continuous contact with the social environment (37) “Yes, well, due to the fact that I’m at home with my family in the mornings and also at other times except for these 3 days, I can continue fulfilling my obligations in the family. It is not as restrictive as it would be if I were in a day-care clinic.”(C) “I’m fine there, even if there might be a lot of problems, but all the same I get the feeling of being safe and having a place to retreat to. And this is exactly what I wouldn’t have in inpatient therapy.”(F)
T1.3.1 Maintaining autonomy and independence (10) ”Well, I wanted to stay in my life. It was important to me to be able to manage independently in my everyday life. So that was one of my goals and, um, I don’t know, I’ve never received inpatient therapy before, but I have the feeling that everything, like preparing food etc., is done for you.”(G) T1.3.2 Promptly implementing therapeutic contents (65) ”Yes, that I have the feeling that I am doing something for my daily routine and, um, I can practice, maybe reorganize my life, maybe a little bit. That was also a concern of mine—to get out of this old rut in which I was stuck” (O) T1.3.3 Continuous contact with the social environment (40) ”[….] in which you can basically keep on with your regular daily routine without being afraid of missing out on something or letting others hang.”(F) ”Yes, of course I simply had to put some people off and say: “sorry, I can’t do it at the moment, I can’t manage it”.(L)

**Table 7:** Quotes on supply needs before (T0) and after (T1) treatment

4. Supply needs
T0.4.1 Intensity of treatment (76) “As I have already said, the fact that my social contacts have decreased—I just don’t feel well, I have problems… I sleep too much, I’m constantly tired and listless, I can’t get my act together. And I think that it helps to be there 3 times a week and work on my problems intensively.”(V) T0.4.2 Multimodality of the treatment offer (32) “I think when you receive outpatient treatment, you just have your one-on-one therapy session once a week. Now, in the evening clinic, there are options that you basically don’t get in the usual outpatient setting: one-on-one sessions, group sessions, mindfulness training and consultations.”(Y) T0.4.3 Less waiting time (16) ”[…] the main thing was to get help quickly, and not have to wait for months to get therapy. This just was the option that was available for me at the time.”(W)
T.1.4.1 Intensity of treatment (73) ”It wouldn’t have worked with outpatient therapy; I’m convinced that wouldn’t have been enough.”(E) T1.4.2 Multimodality of the treatment offer (31) ”[….] I just liked the mixture between the mindfulness training, the consultations with a doctor, the one-on-one therapy sessions with the therapists and the interaction in the group—just the whole package.”(U)

**Table 8:** Quotes on compatibility with professional life before (T0) and after (T1) treatment

5. Compatibility with professional life
T0.5.1 Avoiding being absent from work (51) ”But I’d feel pretty bad if I couldn’t work because of that at the moment. That would beat me up”(C) T0.5.2 Work as a stabilizing factor (11) “And when I work and when I have a job then I’m distracted. I’ve filled my time in a meaningful way. And it also gives me the feeling of independence and of not being helpless. If I’d just sit at home, I’d feel even sicker. Like this, at least I have the feeling of being a part of society.”(G) T0.5.3 Support in job-associated topics (10) “Of course I hope to find a new job relatively soon—one that is possible because of my treatment in the evening clinic. That this is possible at the same time and that I’ll have support here. That would also be an advantage of the evening clinic over in-patient therapy.”(F) T0.5.4 Time challenge (34) “Well, that you come here stressed out, your head is not free, you are still thinking of work or of your kids, that you might overtax yourself even though it is supposed to be a positive offer—but perhaps it will return as a boomerang?”(H)
(G) Avoiding being absent from work (23) ”Well, um, I don’t think I would have come this far if I hadn’t been working.”(A) ”Of course, there’s also a certain fear of what could happen if it became known, yes, of course.”(D) T1.5.2 Support with professional topics (9) ”[….] and then I started vocational rehabilitation, but first I was working part-time and now, slowly, I am beginning to work full-time. And I realize how other aspects in the job are starting to surface again [….]”.(M) T1.5.3 Time challenge (30) ”Being here from 5:00 p.m. three times a week and to juggle this with the job wasn’t always easy.”(B)

#### 1. The therapy process (246)

##### T0.1.1. Tasks and goals (115)

The majority of the participating patients hoped that their symptoms would be rapidly alleviated and that they would receive professional guidance in coping with specific everyday difficulties. They expected to learn strategies that would help them to deal with confusing emotions and mood swings as well as improve their listlessness and depressed mood. Further, patients assumed that the evening clinic would provide them with a safe environment to discuss difficult situations as well as general and private problems, as they found it unpleasant and embarrassing to discuss these issues with friends or family. In addition, some patients wished to regain a day-to-day structure and social contacts through treatment, which would in turn help them feeling less listless or socially isolated.

##### T0.1.2. Difficulties during the therapeutic process (36)

Especially, patients who had previously had no experience with psychotherapy expressed doubts and scepticism regarding psychotherapeutic treatment and its success. They feared that it would be challenging for them to seek outside help, open up during therapy, and to accept feeling vulnerable. Some patients were also concerned that they would be emotionally stressed by engaging with intense therapeutic issues and difficult affects during treatment. Particularly in the beginning of their treatment, patients were afraid that their symptoms would worsen and that this would have negative effects on their professional and psychosocial performance.

##### T1.1.1. Tasks and goals (59)

Overall, many patients found the evening clinic to be a helpful and supportive treatment offer regarding their personal difficulties. These patients experienced the evening clinic as a safe and sheltered place, where they were able to talk about their personal concerns and struggles.

##### T1.1.2. Difficulties during the therapeutic process (36)

However, other patients reported that they had received too little support in the evening clinic and had, therefore, felt abandoned. Especially in the initial phase of treatment, these patients would have liked more guidance and closer assistance from the therapists. This difficulty particularly affected patients who had undergone psychosomatic–psychotherapeutic therapy for the first time.

#### 2. Group experience (753)

##### T0.2.1. Interacting in the group (63)

Before starting with the treatment, many patients said they hoped that the group setting as a protected and familiar environment would give them additional support on the one hand, and provide them with the opportunity to exchange ideas with other affected persons on the other hand. Further, they hoped that gaining new experiences in the group would help them to develop different perspectives on their individual problems and find alternative ways of dealing with them. Many patients expected the combination of the group setting with one-on-one consultations was more beneficial than the usual one-on-one outpatient therapy sessions.

##### T0.2.2. Community experience and feelings of belonging (42)

Some evening clinic patients hoped to connect with other patients and to feel they were not alone with their individual problems. They reported that they had withdrawn from their social surroundings due to their illness, which had led to feelings of inferiority, loneliness, and sadness. In the patients’ perception the evening clinic offered the possibility of meeting people with similar problems, enabling them to experience feelings of community, belonging, and attachment. The patients expected this to be an important advantage of the evening clinic setting over dyadic psychotherapy.

##### T0.2.3. Learning through interactions in the group (28)

Evening clinic patients hoped to improve social communication and interaction skills by engaging with other participants (e.g. self-disclosure, learning to draw personal boundaries). They expected that this would also help them to be more open and relaxed in social interactions in their private life.

##### T0.2.4. The challenge of opening up in the group (79)

At the same time, evening clinic patients feared that being in a group could become challenging and exhausting. They expressed feeling wary about trusting strangers and exposing themselves by talking about their personal problems in this unfamiliar (therapeutic) situation. The idea of showing vulnerability or seeking and accepting help seemed somewhat arduous, unpleasant and embarrassing. On the one hand, patients were afraid of feeling ashamed and disappointed, or of being attacked, insulted and marginalized in the group without being able to protect themselves. On the other hand, they feared that they themselves could unintentionally hurt other group members.

##### T0.2.5. Engaging with the topics of the other group members (11)

Another concern related to the group setting was that patients feared they would not be able to sufficiently distance themselves from other group members and their personal problems. They worried that this would cause them to feel overburdened and they would, thus, benefit less from the whole therapeutic process.

##### T1.2.1. Interacting in the group (107)

After having completed the treatment cycle in the evening clinic, many patients reported that they had benefited a lot from the mutual exchange within the group setting. They found the interactions with fellow patients who had similar problems to be helpful, enriching and unburdening. This exchange opened up new perspectives and created feelings of not being alone with particular struggles. Patients described that it became easier for them to talk about themselves and that they had become more open. This was ascribed to a kind of community feeling in the group setting. Overall, the combination of one-on-one consultations and group therapy sessions was seen as being more beneficial than individual outpatient psychotherapy.

##### T1.2.2. Community experience and feelings of belonging (215)

In retrospect, evening clinic patients experienced their involvement in the patient group and the patient community as something very valuable. The feeling of belonging and being accepted was described as unburdening and empowering. It increased the patients’ self-confidence, self-esteem, and self-acceptance.

##### T1.2.3. Learning through interactions in the group (64)

In addition, many patients experienced and used the group as a training ground to improve their social interaction and communication skills. This was facilitated by the caring environment of the group. Also, some patients described that they had learnt more about their own behavioural patterns which enabled them to engage better with their individual feelings and personal needs.

##### T1.2.4. The challenge of opening up in the group (74)

Some patients reported that it was a major challenge, especially in the early stages of treatment, to build up trust towards other group members and to open up. These patients expressed restraint to talk to strangers about their private issues for fear of feeling embarrassed or being hurt. For newcomers, it was intimidating that other group members who had been participating for a longer time were very familiar with each other.

##### T1.2.5. Engaging with the topics of the other group members (34)

For some patients it, was difficult to engage with the problems of fellow group members while maintaining their personal distance. The intense discussions in the group were perceived as stressful. Patients felt that they would have needed to address these burdens directly after the group session in additional (one-on-one) therapeutic consultations.

##### T1.2.6. The challenge of a semi-open group setting (36)

As the group was organized as a semi-open group, patients had to adapt to a certain fluctuation of group members. Some patients perceived this as challenging because they found that the constant change disturbed the whole group and the process of building up trust.

#### 3. Staying involved in everyday life (214)

##### T0.3.1. Maintaining autonomy and independence (38)

Evening clinic patients hoped to be able to maintain their self-reliance, self-esteem, and self-efficacy in their everyday lives while undergoing treatment in the evening clinic. They regarded the alternative of inpatient therapy and thus being removed from everyday life as a loss of independence and a personal failure. Some patients were not ill enough to be admitted to hospital, yet too ill to attend regular low-frequency outpatient treatment. Thus, the evening clinic could combine the need for intensive treatment while, enabling patients to feel self-sufficient at the same time.

##### T0.3.2. Promptly implementing therapeutic contents (24)

The patients saw the evening clinic setting as an opportunity to discuss their current everyday struggles in therapy and promptly practice dealing with them in their daily life (transfer aspect). They hoped that this would facilitate their discharge at the end of treatment and prepare them for their return to their unassisted everyday life.

##### T0.3.3. Continuous contact with the social environment (37)

Patients who were well integrated in their social environment often experienced this as supportive and stabilizing, especially during illness. Therefore, these patients expected that keeping in contact with their home environment or with their family and friends during treatment would be beneficial. A few patients also had private obligations, such as caring for children or relatives, which prevented inpatient treatment. Therefore, these patients hoped to be able to continue carrying out their duties while undergoing treatment in the evening clinic. In addition, some patients expected it to be favourable that, unlike inpatient treatment, attendance of the evening clinic could be kept a secret from relatives or acquaintances in order to avoid feeling uncomfortable or burdening others who had their own problems. However, patients who had very busy private lives expected making time to visit the evening clinic to be demanding. Others even feared feeling guilty towards their families due to their regular evening absences.

##### T1.3.1. Maintaining autonomy and independence (10)

After having completed the treatment, many patients reported that they perceived it as very valuable that their attendance in the evening clinic had not restricted their personal independence. These patients were socially and professionally well integrated and able to care for themselves. A removal from the familiar environment would have been experienced as a failure and incapacitation. Thus, the preservation of independence and self-efficacy helped patients to strengthen their feelings of self-esteem and reduce their subjective malaise.

##### T1.3.2. Promptly implementing therapeutic contents (65)

Evening clinic patients experienced it as very helpful to be able to introduce topics from their everyday lives into the group discussions. In retrospect, many patients also benefited from the opportunity to integrate issues that had been talked about during therapy into their everyday lives. They could practice implementing certain aspects in between the individual evening clinic sessions. As a result, the therapy in the evening clinic was experienced as being closer to everyday life and more “real”, which in turn facilitated the patients’ parting at the end of their treatment.

##### T1.3.3. Continuous contact with the social environment (40)

In hindsight, many patients appreciated the ability to remain in their familiar social environments during treatment which meant that they could continue to pursue social obligations and everyday habits. The close contact with their usual surroundings had a stabilizing effect and provided security for the intensive treatment in the evening clinic. For many patients, it was either unimaginable or impossible that they would be removed from their surroundings in order to undergo inpatient treatment due to everyday obligations. However, some patients also reported restrictions in their private lives as a result of the time-consuming treatment in the evening clinic. This often was accompanied by feelings of guilt towards relatives. Yet, because treatment in the evening clinic took place within a limited period of time, patients were able to accept these circumstances more easily.

#### 4. Supply needs (228)

##### T0.4.1. Intensity of treatment (76)

Patients expected that the evening clinic would provide them with intensive, high quality treatment in a short period of time. Compared to regular outpatient therapy, they hoped that the high frequency of treatment would translate into a better chance of success. Some patients also hoped that they would recover from their illness faster and, thus, be able to shorten the time of therapy. Other patients thought that the comprehensive therapy was necessary due to their perception of personal impairment or as a starting-point for further, less frequent outpatient therapy. Therefore, most patients considered the high intensity of the evening clinic setting as an advantage.

##### T0.4.2. Multimodality of the treatment offer (32)

The evening clinic consisted of several different treatment offers: individual and group therapy, family and couple therapy, mindfulness training, and consultations with a social worker. This resulted in patients ascribing a higher quality of treatment and better care to the evening clinic setting. Thus, their general expectations concerning the efficacy of the treatment compared to outpatient treatment were greater.

##### T0.4.3. Less waiting time (16)

In addition, patients who had previously experienced long waiting lists for outpatient treatment during acute crises cherished the faster admissions and flexible treatment offers of the evening clinic.

##### T1.4.1. Intensity of treatment (73)

After having undergone treatment in the evening clinic, patients rated the intensity of the treatment as positive. They reported that this therapeutic approach had encouraged them to continue working at specific issues, a fact that caused them to regard the treatment as being more efficient. According to many patients, outpatient psychotherapy would not have been sufficient or would have taken too long. In several cases, the intensive evening clinic treatment represented the starting point for less frequent outpatient psychotherapy. However, other patients found the high intensity of treatment in the evening clinic to be challenging and quite demanding.

##### T1.4.2. Multimodality of the treatment offer (31)

The overwhelming majority of evening clinic patients reported benefiting from the combination of different group offers. They experienced the wide range of therapeutic options as being more efficient for the whole recovery process than other, unimodal treatment procedures.

#### 5. Compatibility with professional life (168)

##### T0.5.1. Avoiding being absent from work (51)

Many patients expected the option of being able to continue with their professional lives while being treated in the evening clinic to be an advantage. These patients were well integrated in their workplaces and experienced themselves to be functioning sufficiently in a professional context. They stated that on the one hand they felt a sense of obligation towards their colleagues and employers, and on the other hand they feared financial difficulties, disclosure, stigmatization or other negative consequences in the case of a longer absence from work.

##### T0.5.2. Work as a stabilizing factor (11)

Some patients hoped the combination of continuing with their professional careers while being treated in the evening clinic to be a stabilizing and self-reinforcing experience. They expected to be able to maintain their independence and self-efficacy during the treatment, and they also hoped that the parallel engagement with other topics would alleviate their subjective malaise.

##### T0.5.3. Support with professional topics (10)

Patients with a current incapacity to work while undergoing treatment hoped for support in vocational reintegration, i.e. receiving help in searching for jobs, reorienting themselves professionally or clarifying specific issues of labour law.

##### T0.5.4. Time challenge (34)

At the same time, evening clinic patients who were currently in employment feared the coordination of therapy and work to be a challenge. Working parents of younger children found the idea difficult to be absent from home on three evenings a week because it meant that they saw their children even less or could not support their partners in childcare. Patients who were already impaired due to their illness were worried that treatment would put them under more pressure which would lead to further symptom deterioration rather than improvement.

##### T1.5.1. Avoiding being absent from work (23)

After the completion of treatment, patients stated that the evening clinic gave them the opportunity to continue working while undergoing therapy. Thus, they did not have to give up their professional integrity. Furthermore, continuing to work while also caring for their health gave patients an increased feeling of self-esteem and self-efficacy. They found it important to avoid being absent from work due to a sense of duty towards colleagues, concerns of negative consequences from the employer as well as loss of face or stigmatization. Some patients also feared financial shortages due to a prolonged sick leave and expiry of unemployment insurance benefit. In general, however, it was unproblematic for patients to discuss the need of adapting their working hours to the times of the evening clinic with their employers.

##### T1.5.2. Support with professional topics (9)

In addition, evening clinic patients found the support by therapists and social workers concerning professional issues helpful and stress-relieving. Typical topics were: what kind of assistance they could receive once they returned to work and how they could go about reintegration, job search or preparing for job interviews.

##### T1.5.3. Time challenge (30)

At the same time, the double workload and intensive evening-clinic treatment were experienced as a challenge and sometimes even as a burden, especially for patients working full-time. Patients who had to travel some distance to the evening clinic struggled to coordinate their time and spent most of their days away from their homes. However, the patients were prepared to accept this additional burden in order to be able to attend therapy in the evening clinic.

## Discussion

In the present qualitative study, five categories were formed from the evening clinic patients’ statements in the interviews. These are related to (1) aspects of the therapeutic process, (2) patients’ experiences concerning the group setting, (3) the possibility to stay involved in everyday life, (4) supply needs, and (5) the compatibility of treatment in the evening clinic with patients’ professional life. Altogether, the statements of the patients were quite similar before and after treatment in the evening clinic. In the following paragraphs, the five categories will be discussed separately. Our particular interest lies in investigating the patients’ point of view on parallels and differences as well as advantages and disadvantages of the evening clinic compared to other outpatient treatment facilities, partial inpatient treatment services and inpatient psychotherapy.

### 1. Aspects of the therapeutic process

The patients’ expectations before starting treatment (T0) mainly concerned functional areas, such as their daily functional performance, problems of everyday life in general, family relationships, social environment, and rebuilding professional skills. Clinical experience and qualitative research with patients undergoing outpatient treatment, day care treatment or inpatient treatment have shown similar treatment goals. International studies show, that outpatients with depressive disorders hoped that therapy would improve their social and family relationships, health, professional lives and their structure in everyday life [38, 39]. In the current study, most patients who had completed treatment in the evening clinic (T1) stated that they had experienced the whole therapy as helpful and supportive to improve their symptoms and be able to deal with everyday life problems. These aspects also can be found in others settings and seem to be independent of the concept and structure of the evening clinic [40]. However, especially patients without prior experience of psychotherapy expressed doubts before starting treatment (T0) whether they would benefit from therapy in the evening clinic. They were concerned that they would not be able to work on their personal difficulties sufficiently. These fears seem to be linked specifically to the finding that on the one hand patients have higher expectations towards the evening clinic, and on the other hand they are aware of the fact that this setting offers lower treatment intensity compared to inpatient and day-care hospital treatments. As a result, some of the patients who were in need of intensive care were disappointed in the evening clinic setting and felt insufficiently supported. It remained unclear whether the criticism also was due to uncertainty towards this novel concept, or whether it was based on needs that cannot be met in the evening clinic setting and would lead to a stricter process of deciding which patients are suitable for treatment in the evening clinic.

### 2. Patients’ experiences concerning the group setting

Regarding the group setting, we found some parallels and some differences in the patients’ comments compared to established treatment settings. Interestingly, the group setting in the evening clinic played a major role for the patients, both before and after their treatment. Combining individual therapy sessions with group therapy, appealed to the patients and was expected to be more beneficial than the usual individual therapy in outpatient settings. Interacting with other patients in the group, experiencing feelings of community, encountering people with similar problems, and improving their social skills were stated as benefits. Similarly, in a qualitative study by Nikendei et al., day-care patients and inpatients thought that social aspects of the group setting, such as interacting with other people, practicing social competences, and experiencing a sense of belonging, were important therapeutic components [40]. In addition, the fears concerning the group setting, such as opening up and dealing with the problems of other group members are similar to the anxieties that patients of the evening clinic reported [40]. However, especially patients without prior experience of psychotherapy found the concept of an open group and the unsettlement caused by a certain fluctuation of patients in the group difficult, as it meant that they had to enter an ongoing therapeutic process. This new aspect was stated by many evening clinic patients and did not occur in day-clinic or inpatient research [30].While there are open groups with regular changes of group members in most day-care and inpatient therapy settings, outpatient groups are mostly closed. There could be different reasons for evening clinic patients finding the unstable group situation difficult: Perhaps they had more expectations in the group due to the group-based setting of the evening clinic, causing patients to feel particularly sensitive when the group cohesion was disturbed. A further reason could be that patients of the evening clinic are affected by the usual fluctuations and changes in other areas of everyday life and therefore there is already much unrest in their lives. Based on previous research on the importance of group cohesion within a therapeutic community [41–43] our findings call for special attention to the patients’ integration.

In inpatient treatment facilities, group therapy is one of the common therapeutic elements and its efficacy for different mental disorders has been studied in detail [44]. The results of a meta-analysis highlight the valuability and effectiveness of group therapy in inpatient setting by comparing a patient cohort receiving group therapy with a control group receiving one-on-one therapy or being on the waitlist [45]. Other studies have shown that group therapy is also effective in outpatient settings [46]. Our results show, that by combining group therapy elements and individual therapy sessions in an outpatient evening-clinic setting, evening clinic seem especially favourablefor patients who have sufficient resources to form strong relationships and build up trust, while not being emotionally too involved.

### 3. Staying involved in everyday life

The interviewed patients perceived the close relationship of therapy and everyday life as an advantage compared to inpatient treatment programs. Before as well as after treatment (T0 and T1), patients stated it to be supportive and stabilizing to continue being autonomous and self-determined in their familiar environments and fulfil their private and professional obligations while undergoing therapy. Patients interviewed before treatment expected it to be helpful to be able to transfer aspects from therapy into their daily lives and vice versa. Patient interviewed after treatment answered in similar way and experienced it also helpful to facilitate their return to their unassisted daily lives after completing therapy in the evening clinic. In day-care treatment settings there is a similar combination of intensive therapy and normal everyday routine and studies assessing patients’ perceptions on day-care treatment show similar results. As described in the study by Nikendei et al., patients found it reassuring that they were still integrated into their social environments [40]. They stated that communication in the family had improved as a result of promptly integrating issues discussed in therapy into their daily lives. Patients experienced their eventual discharge from therapy to be easier due to the interconnectedness of everyday life and therapy [40].

As described in a study by Zeeck et al., patients who are treated in day-care facilities show an improved daily transfer compared to full-time patients [13]. According to Mörtl et al., the successful integration of therapeutic content and skills into everyday life is an important factor in day-care treatment concepts [47]. Other studies show that in particular those patients who live in a partnership or have family seem to benefit from day-care treatment settings [48]. Thus, compared to inpatient therapy, day-care psychotherapy is not only less expensive, but also has benefits concerning many other aspects [49]. As our study shows, these factors also seem to play a role in the evening clinic setting.

However, similar to day-clinic treatment, the results of the current study show that the co-ordination of intensive psychotherapeutic treatment and regular daily life was often experienced as challenging, exhausting and intense. In addition, some patients felt overwhelmed by therapy and would have needed more support. Obviously, the concept of an evening clinic carries the risk of patients overestimating themselves, especially when they are working. It is likely that the evening clinic is particularly challenging for depressive patients, as loss of energy represents one of the major diagnostic criteria [50]. In their non-randomized, observational INSTAP study, Zeeck et al. found a significant negative relationship between loss of energy and clinical outcome for day clinic patients [9]. Therefore, the question whether or not a significant loss of energy is a specific hindrance also for evening clinic therapy should be addressed in the preliminary discussions and taken into account during treatment. Especially patients who have little personal resources and coping skills in everyday life need a more intensive therapeutic support, possibly entailing more frequent consultations than the evening clinic model allows for. Therefore, the evening clinic concept includes times for dinner and mindfulness exercises in the treatment plan.

### 4. Supply needs

Many of the patients we surveyed expected an intensive, high-frequency, and efficient therapy (T0). They hoped for treatment success due to professional and multimodal care. After treatment in the evening clinic (T1), many participants reported that they had experienced the multimodal concept and the high frequency of therapy as very helpful. Inpatient and day-care treatment programs already use multimodal treatment concepts that combine verbal and non-verbal therapy methods in individual and group settings [51, 52]. By contrast, according to the guidelines in the German healthcare system in outpatient settings, multimodality is currently not possible. Overall, the observations from the T0 and T1 interviews show the patients’ interest in intensive treatment with elements of inpatient treatment concepts, while also drawing from the benefits of outpatient guideline psychotherapy. In our study, we noticed a certain idealization of treatment in the evening clinic. This seems to contribute to patients’ appreciating the therapeutic content to a larger extent and showing higher motivation to participate in group discussions or activities and can lead to an intensive group process [23]. On the other hand, this aspect also can represent a risk for disappointment.

### 5. Compatibility with professional life

In average, evening clinic patients were younger than day-care patients or inpatients [14]. Compared to inpatient programs, a high proportion of male patients were interested in the evening clinic. One possible reason could be the finding that male patients seemed more afraid than women of stigmatization due to inpatient treatment, so that the opportunity to continue working in their jobs while undergoing therapy seemed especially appealing [23]. The majority of the patients surveyed were working or studying. A smaller part of the cohort planned their professional reintegration during or after having completed treatment in the evening clinic. For many patients the compatibility of treatment in the evening clinic with their professional lives played an important role. These factors resulted in a large amount of therapy time being spent on topics related to work and it was experienced as a particular advantage of the evening clinic’s treatment offer.

Outpatient treatment settings also enable patients to continue working in their jobs, whereas in day-care and inpatient treatment settings this usually is not possible. Due to the fact that the evening clinic provides more intensive therapy than is usual in outpatient treatment, patients found it difficult to combine the evening clinic with their professional lives. This was stated by many patients before and after treatment (T0 and T1) and indicates the necessity for a high degree of commitment and responsibility, combined with self-reliance, motivation and conscientiousness. It requires a certain degree of coping and efficiency in everyday life as well as sufficient resources for patients to be able to juggle all obligations. This aspect seems to represent the most specific difference of evening clinic treatment as an independent concept in the field of tension between outpatient and inpatient treatment. There is also a risk that patients who over-estimate themselves might prefer treatment in the evening clinic even though hospitalization would be indicated due to the severity of their symptoms. This aspect should receive special attention by the therapists who can support patients, for instance by suggesting short-term sick leave in the beginning of the treatment cycle.

### 6. Contrasting analysis of the most frequent topics before and after the treatment

The reported topics before and after treatment show many similarities. This may be largely explained by the fact that the same participants were interviewed before and after treatment in the evening clinic. However, we also found some differences. First of all, the compatibility of evening clinic treatment with professional life seems to be important for the participants before treatment due to a sense of responsibility, feelings of guilt or financial concerns. After treatment, fewer participants emphasised professional topics. This finding may be explained by a change in self-expectations and perceived inner pressure during the therapy.

In contrast, more participants reported they found the group experience very important after treatment, especially the community experience and feeling of belonging. This suggests that patients that had initially felt insecure ore indifferent in group therapy were able to make different experiences.

Furthermore, participants only reported to find the semi-open group setting challenging after treatment in the evening clinic. This can be explained by the fact that few patients had experience with a semi-open group setting before starting treatment. Furthermore, two topics were unique to the evaluations before treatment, i.e. the short waiting time and the experience of work as a stabilizing factor. This suggests that participants experience a lower level of psychological burden after than before treatment, which may explain why the first point was less important to them. The second point might be explained by the fact that the evening clinic has taken over the stabilizing effect.

Overall, the results of the current study show that patients treated in the evening clinic want to remain in their private and professional environments on the one hand, and on the other hand need intensive psychotherapy. The evening clinic model seems to offer a compromise acceptable to many. Mental illness is the second leading cause of disability in Germany and in case of illness patients have the longest periods of sick-leave [53]. In this respect, evening clinic treatment could offer a possibility of early intensive psychotherapeutic intervention while still being able to continue working and thus might prevent or shorten sick-leave. Our study is limited by the small number of participants due to its qualitative approach. As the entire spectrum of psychosomatic diseases from depression and anxiety disorders to somatoform disorders is treated in the evening clinic presents heterogeneous diagnose. Another limiting factor was that the study was performed in qualitative design and, thus, does not provide any indication of the efficacy and effectiveness of the treatment. Furthermore, although the qualitative content analysis was performed according to principles of inductive category development, the examination can be considered to be less generalizable than quantitative approaches due to the subjective nature of qualitative studies. However it should be noted that the main aim of this study was to identify specific characteristics of the evening clinic setting that were perceived as especially helpful or adversely and not to study the therapeutic effectiveness. Therefore, this methodical approach was specifically chosen in order to provide a more complete picture of the patients’ subjective experience and to identify new, distinct aspects regarding this treatment setting. Our study shows, that treatment in the evening clinic is particularly suitable for patients with a certain level of stability in their daily lives and sufficient resources to avoid being overworked. To investigate this more closely, psychometric studies of the treatment effects of the evening clinic concept would be needed and are currently running in our university.

## Conclusions

The concept of an evening clinic is beneficial for patients with mental and psychosomatic illnesses. It was well received by the patients in the pilot study. Our results indicate that treatment in an evening clinic is particularly suitable for patients who on the one hand have a need for psychotherapy that goes beyond the usual outpatient treatment, and on the other hand still have sufficient personal resources and are well integrated in their private and professional lives. Most of these patients would like to avoid hospitalization which would mean that they would be removed from their social and professional environments. Thus, evening clinic treatment was experienced as an opportunity to perform intensive multimodal psychotherapy while maintaining social and professional integrity and autonomy. Consequently, this therapeutic concept offers the possibility of reducing costs in the healthcare system. In order to be able to discuss these aspects in more detail, further research projects are planned to investigate the treatment effects and the exact cost-effectiveness of an evening clinic treatment.

## Data Availability

The datasets generated and analyzed during the current study are available from the corresponding author on reasonable request.
